# Effect of cognitive reserve on amnestic mild cognitive impairment due to Alzheimer’s disease defined by fluorodeoxyglucose-positron emission tomography

**DOI:** 10.3389/fnagi.2022.932906

**Published:** 2022-08-10

**Authors:** Takashi Kato, Yukiko Nishita, Rei Otsuka, Yoshitaka Inui, Akinori Nakamura, Yasuyuki Kimura, Kengo Ito, SEAD-J Study Group, Hidenao Fukuyama

**Affiliations:** Kyoto University, Kyoto, Japan; Institute of Biomedical Research and Innovation, Kobe, Japan; Tokyo Metropolitan Institute of Gerontology, Tokyo, Japan; Kindai University, Osaka, Japan; Kobe Gakuin University, Kobe, Japan; Kobe University Graduate School of Medicine, Kobe, Japan; Hamamatsu University School of Medicine, Hamamatsu, Japan; Kizawa Memorial Hospital, Gifu, Japan; National Center for Geriatrics and Gerontology, Aichi, Japan; National Center for Geriatrics and Gerontology, Aichi, Japan; Tohoku University Graduate School of Medicine, Sendai, Japan; Nagoya University School of Health Sciences, Nagoya, Japan; ^1^Department of Clinical and Experimental Neuroimaging, National Center for Geriatrics and Gerontology, Aichi, Japan; ^2^Department of Epidemiology of Aging, National Center for Geriatrics and Gerontology, Aichi, Japan; ^3^Department of Radiology, Fujita Health University School of Medicine, Aichi, Japan; ^4^Department of Biomarker Research, National Center for Geriatrics and Gerontology, Aichi, Japan

**Keywords:** cognitive reserve, mild cognitive impairment, Alzheimer’s disease (AD), cerebral glucose metabolism, education

## Abstract

This study aimed to investigate the effect of cognitive reserve (CR) on the rate of cognitive decline and cerebral glucose metabolism in amnestic mild cognitive impairment (MCI) using the Study on Diagnosis of Early Alzheimer’s Disease-Japan (SEAD-J) dataset. The patients in SEAD-J underwent cognitive tests and fluorodeoxyglucose-positron emission tomography (FDG-PET). MCI to be studied was classified as amnestic MCI due to Alzheimer’s disease (AD) with neurodegeneration. A total of 57 patients were visually interpreted as having an AD pattern (P1 pattern, Silverman’s classification). The 57 individuals showing the P1 pattern were divided into a high-education group (years of school education ≥13, *N* = 18) and a low-education group (years of school education ≤12, *N* = 39). Voxel-based statistical parametric mapping revealed more severe hypometabolism in the high-education group than in the low-education group. Glucose metabolism in the hippocampus and temporoparietal area was inversely associated with the years of school education in the high- and low-education groups (*N* = 57). General linear mixed model analyses demonstrated that cognitive decline was more rapid in the high-education group during 3-year follow-up. These results suggest that the cerebral glucose metabolism is lower and cognitive function declines faster in patients with high CR of amnestic MCI due to AD defined by FDG-PET.

## Introduction

In aging and dementia, the concept of cognitive reserve (CR) is associated with the capacity of the brain to cope with neuropathology in order to minimize clinical manifestations (Stern, 2002). According to the CR hypothesis, in advanced degenerative dementia, individuals with a high CR should have more advanced dementia pathology than those with low CR if they have the same level of cognitive function, which has been proven in a number of studies (Lovden et al., 2020).

Stern’s CR hypothesis includes another important claim wherein he predicted that individuals with a high CR would retain cognitive function despite pathology; however, once the decline begins, the rate of decline would be faster than that in individuals with a low CR. Nevertheless, the results from various research groups have been inconsistent. Compared with low CR, cognitive decline in high CR has been reported to be faster (Unverzagt et al., 1998; Andel et al., 2006; Scarmeas et al., 2006), slower (Fritsch et al., 2001; Hall et al., 2007; Bennett et al., 2015), or similar (Del Ser et al., 1999; Paradise et al., 2009; Vemuri et al., 2011). One possible reason for this discrepancy could be attributed to the varying etiologies and disease stages among the reports. In recent years by using imaging or liquid biomarkers, it has become possible to optimize examinations of CR for Alzheimer’s disease (AD) related clinical progression across AD continuum (McKhann et al., 2011), including preclinical AD, amnestic mild cognitive impairment (MCI) due to AD, and AD dementia (van Loenhoud et al., 2019; Lee et al., 2021).

This study aimed to investigate the effect of CR on the rate of cognitive decline and cerebral glucose metabolism in amnestic MCI due to AD using data from the Study on Diagnosis of Early Alzheimer’s Disease-Japan (SEAD-J) (Ito et al., 2015; Inui et al., 2017). The SEAD-J was a prospective study to investigate the predictive ability of fluorodeoxyglucose-positron emission tomography (FDG-PET) in the conversion from amnestic MCI to dementia in 3-year follow-up. Visual reading of the pattern of decreased glucose metabolism on FDG-PET at the baseline was employed to narrow down the included participants to MCI due to AD. Neurodegenerative progression was estimated based on the degree of cerebral glucose hypometabolism.

## Patients and methods

### Patient information

#### Study on Diagnosis of Early Alzheimer’s Disease-Japan

The data used in this work were retrieved from the SEAD-J database. The SEAD-J was launched in 2005 as a multicenter (nine institutions in Japan) cohort study of amnestic MCI. The primary intent of the SEAD-J was to establish scientific evidence for the usefulness of imaging biomarkers (F-18 FDG-PET) in the early diagnosis of AD at the stage of amnestic MCI. Clinical and neuropsychological assessments can be combined to evaluate amnestic MCI progression.

A total of 114 patients (64 women and 50 men; mean age, 70.8 ± 7.5 years) were enrolled in the SEAD-J. Notably, 88 patients reached the endpoint (conversion to dementia) or completed the 3-year follow-up. In this study, 57 out of the 88 patients were examined whose FDG-PET images were visually interpreted as an AD pattern (P1, described in the visual interpretation of the FDG-PET images), respectively. The 57 individuals showing the P1 pattern were divided into a high-education group (years of school education ≥13, *N* = 18) and a low-education group (years of school education ≤12, *N* = 39) (Table 1 and Supplementary material). In the survey and statistical analysis model of this study, years of school education was specified as a discrete variable. In the Japanese schooling system, as well as in the United States, 13 or more years of education usually implies some form of education beyond secondary school. The subjects were stratified into two groups using the separating point for years of education while considering the distribution of the educational attainment and the number of the subjects.

**TABLE 1:** Demographic characteristics of the patients at the baseline and conversion rate in a 3-year follow-up.

	Total	Low-education	High-education	*p*
N	57	39	18	
Age	71.9 (6.4)	73.1 (5.2)	69.3 (8.0)	0.054
Sex (male/female)	28/29	14/25	14/4	0.001
Years of school education	11.6 (2.9)	9.9 (1.6) (≤12)	15.1 (1.5) (≥13)	<0.001
CDR	0.5	0.5	0.5	
MMSE	25.6 (1.8)	25.8 (1.7)	26.0 (1.6)	0.826
ADAS-Jcog	10.1 (5.0)	10.5 (5.5)	9.2 (3.5)	0.294
WMS-R LM-I	7.3 (3.2)	7.3 (3.3)	7.3 (3.0)	0.930
WMS-R LM-II	2.2 (2.5)	2.2 (2.5)	2.4 (2.5)	0.677
GDS	4.4 (2.3)	4.2 (2.4)	4.8 (2.0)	0.569
Converter/non-converter	35/22	22/17	13/5	0.442

Values are presented as mean (standard deviation) or number of participants. Differences in sex and conversion/non-conversion between the high and low cognitive reserves were tested using the chi-square test. Other continuous parameters were tested using the Student’s *t*-test. CDR, Clinical Dementia Rating Scale; MMSE, Mini-Mental State Examination; ADAS-Jcog, Alzheimer’s Disease Assessment Scale-Cognitive Component-Japanese version; WMS-R LM-I, Wechsler Memory Scale-Revised, logical memory I; WMS-R LM-II, Wechsler Memory Scale-Revised, logical memory II; GDS, Geriatric Depression Scale.

The diagnosis of amnestic MCI was based on an interview with neurologists that revealed evidence of reduced cognitive capacity, normal activities of daily living, and absence of dementia. All the patients were free of significant underlying medical, neurological, or psychiatric illnesses. All the patients were initially assessed using a series of neuropsychological tests, including the Mini-Mental State Examination (MMSE), Alzheimer’s Disease Assessment Scale-Cognitive Component-Japanese version (ADAS-Jcog), Clinical Dementia Rating (CDR), Geriatric Depression Scale (GDS), Everyday Memory Check List (EMCL), and Logical Memory Subset of the Wechsler Memory Scale Revised (WMS-R LM). In accordance with the inclusion criteria, patients with MCI were between 50 and 80 years of age, with an MMSE score ≥24, GDS score ≤10, WMS-R LM-I score ≤13, WMS-R LM-II part A and part B scores ≤8 (maximum = 50), and a CDR memory box score of 0.5. Patients with less than 6 years of formal education were excluded. Each patient signed an informed consent form after receiving full explanation of the procedures involved.

Patients were assessed at 1-year intervals for 3 years. CDR, MMSE, EMCL, and WMS-RLM tests were readministered at each visit. Conversion to dementia was determined when CDR was ≥1.0. No further follow-up of patients with a CDR ≥1.0 was performed. AD was diagnosed when a patient fulfilled both CDR ≥1.0 and the National Institute of Neurological and Communicative Disorders-Alzheimer’s Disease and Related Disorders Association (NINCDS-ADRDA) “probable AD” criteria. Other diseases were diagnosed based on the established clinical criteria, including vascular dementia, dementia with Lewy bodies, and frontotemporal dementia. Two researchers in the SEAD-J group, blinded to the PET results, established the final clinical outcome of each patient based on the submitted case reports. Patients diagnosed with cognitive impairment other than AD, MCI, or normal were excluded from this study.

This study was approved by the ethics committees of all the participating institutions. All participants provided informed consent in accordance with the ethics committee of the National Center for Geriatrics and Gerontology. All the datasets of the clinical and FDG-PET findings over a follow-up period of 3 years were acquired.

### F-18 fluorodeoxyglucose-positron emission tomography acquisition

F-18 fluorodeoxyglucose-positron emission tomography (FDG-PET) scan was performed with the patient in the resting state for 40–60 min following a venous injection of 18F-FDG (254 ± 107 MBq). A static scan was performed for 10 ± 5 min, either in the two or three-dimensional mode. Attenuation was corrected using either a transmission scan with segmentation for dedicated PET or a computed tomography (CT) scan for PET/CT. F-18 FDG-PET images were processed to produce 3D-stereotactic surface projection (SSP) and generate z-score maps using iSSP (version 3.5; Nihon Medi-Physics, Tokyo, Japan). 3D-SSP was created using the Neurological Statistical Image Analysis Software (NEUROSTAT) developed by Minoshima et al. (1995). NEUROSTAT anatomically normalizes the individual PET data to the standard brain and compares the regional voxel data with the normal database, calculating the z-score (| normal mean - individual value| /normal standard deviation) for each voxel of the cerebral surface, and displays the sites at which the voxel value is statistically reduced. The normal database was constructed using 50 control participants (31 men and 19 women; mean age 57.6 years), with 10 participants from each of the five participating institutions. The results of their neurological and brain imaging examinations [magnetic resonance imaging (MRI) or CT] were normal, and their cognitive function was judged to be normal by experienced neurologists.

### Visual interpretation of the fluorodeoxyglucose-positron emission tomography images

Three experts, blinded to the clinical information, independently assessed the reconstructed PET images. They visually evaluate the 3D-SSP z-score maps to classify the images into different dementia patterns according to Silverman’s criteria (Silverman and Phelps, 2001). Based on these criteria, FDG-PET findings are classified into seven interpretive patterns as positive (P1–P3, P1 +) or negative (N1–N3) for the presence of a progressive neurodegenerative disease, in general, and AD specifically compared with the results of longitudinal or neuropathological analysis. P1 indicates progressive PET patterns consistent with the presence of AD showing hypometabolism in the parietal/temporal ± frontal cortex, while P1 +, P2, and P3 indicate progressive PET patterns but inconsistent with AD (P1 +, the presence of abnormal findings other than P1; P2, frontal predominant hypometabolism; P3, hypometabolism of both the caudate and lentiform nuclei). N1–N3 patterns indicate all the negative scans, wherein N1 represents normal metabolism. When the classification of the three raters did not match, a consensus was reached upon discussion of the cases. They referred to the patients’ MRI images to exclude the partial volume effect attributed to severe atrophy, cerebral vascular lesions, and space-occupying lesions when a local hypometabolic area was observed in the PET image. Patients with patterns other than P1 pattern were excluded from this study.

### Magnetic resonance imaging

All the participants were scanned using either a 1.5 T or 3T MRI system. T1-, T2-, and fluid-attenuated inversion recovery-weighted MR images were acquired to rule out neurological diseases, such as cerebrovascular diseases and brain tumors other than neurodegenerative dementia.

### Statistical analyses

For analyses of baseline profiles, we used independent-sample *t*-tests to assess the differences in clinical and cognitive variables, age, years of school education, scores of cognitive tests, and PET scores. The chi-squared test was used for the analyses of sex and age differences between the high- and low-education groups. The chi-square test was also used to determine group differences in the ratio of AD conversion (AD converters vs. non-converters; stable MCI) within the 3-year period between the high- and low-education groups.

General linear mixed models (GLMMs) (Laird and Ware, 1982; Morrell et al., 2009) were employed to evaluate the effects of years of school education on the cognitive changes over a 3-year period. As GLMMs can model differences between groups as random effects, they provide a wide range of models for the analysis of grouped data. GLMMs can handle missing data more appropriately than traditional regression analysis and repeated-measures analysis. The correlations between the repeated measures are adequately accounted for by the variance-covariance structure of random effects. These models are useful for the analysis of many types of data, including longitudinal data. Therefore, a GLMM was chosen for the analysis of intellectual change in some recent studies (Nishita et al., 2019).

The model used in the current study included fixed terms for the intercept (baseline performance for an individual with value zero for all predictors), education (0 = low-education group, 1 = high-education group), time (time in years since baseline), and an education × time interaction term. Age (at baseline) and sex (0 = male; 1 = female) were included as covariates. The random effects of the intercept (baseline performance) and slope (change over time) were calculated using an unstructured covariance matrix. The term of primary interest for this study was the education × time interaction, which reflects whether the high- or low-education groups differ in the rate of change in the cognitive scores, such as the MMSE, ADAS-Jcog, and WMS-R LM I and II scores over time.

Statistical analyses were performed using SPSS Statistics V.17.0 (IBM, Armonk, NY, United States) and SAS System version 9.3 (SAS Institute, Cary, NC, United States). Statistical significance was set at *p* < 0.05.

### Statistical parametric mapping analysis

For voxel-based analyses of group comparisons and multiple regression using SPM8, each FDG-PET image was spatially deformed to the Montreal Neurological Imaging template (PET.nii) using parameters derived from that image and then normalized for variations in whole-brain measurements using proportional scaling. Post-processed images were smoothed to a spatial resolution of 8 mm full width at half maximum. Voxel-based group comparisons were made between high-education and low-education groups with adjustment for the ADAS-Jcog score. Linear regression models were used to evaluate the effect of years of education on cerebral glucose metabolism by adjusting for the ADAS-Jcog score. ADAS-cog score is more precise in measuring the severity of total cognitive dysfunction than MMSE (Balsis et al., 2015). The level of significance was set at *p* < 0.01 and extent threshold >400 (uncorrected). In addition, a region of interest (ROI)-based analysis for FDG-PET data was performed using the MarsBar toolbox.^1^ ROI was created from the statistical map of the regression analysis. This analysis can be a kind of circular statistics (Kriegeskorte et al., 2009), but it only aimed at estimating the strength of the linear relationship between the years of school education and the regional glucose metabolism.

## Results

Table 1 presents the patients’ demographic characteristics at baseline and conversion ratio during the 3-year follow-up. There were significant differences in sex (*p* = 0.001) and years of school education (*p* < 0.001) at baseline between the high- and low-education groups. The differences in age were not statistically significant (*p* = 0.054). The high-education group tended to be younger and included more men than the low-education group. No significant differences were detected between the high- and low-education groups in the CDR, MMSE, ADAS-Jcog, WMS-R LM-I, WMS-R LM-II, and GDS scores. No significant difference in the ratio of conversion to dementia was detected between the high- and low-education groups (Table 1).

**FIGURE 1:**
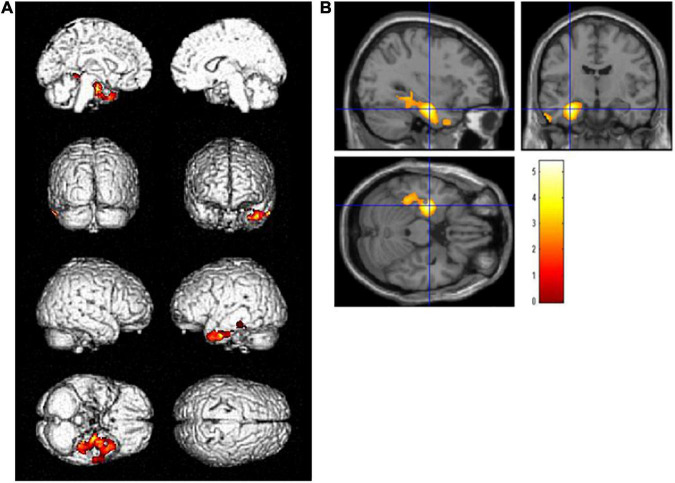
Regions with statistically significant decrease (*p* < 0.01, extent threshold >400) in glucose metabolism PET after adjusting for Alzheimer’s Disease Assessment Scale-Cognitive Component-Japanese version in the high-education group (years of school education ≥13, *N* = 18) compared with the low-education group (years of school education ≤12, *N* = 39) on **(A)** the brain surface projection and **(B)** multi-planar cross-sectional views. The red-yellow scale indicates the level of statistical significance. The blue crossed lines indicate the maximum peak voxel in the left hippocampus [(–32, –11, –23), *T* = 5.40].

A group comparison of FDG-PET images demonstrated that patients with high-education (*N* = 18) had significantly lower glucose metabolism than those with low-education (*N* = 39) in the left hippocampus [(−32, −11, −23), *T* = 5.40] and its surrounding area, including the amygdala, fusiform gyrus, and lingual gyrus, adjusted for ADAS-Jcog [*p* < 0.01 and extent threshold >400 (uncorrected)] (Figure 1). The cluster of the left hippocampus reached a significant level of family-wise error <0.05.

**FIGURE 2:**
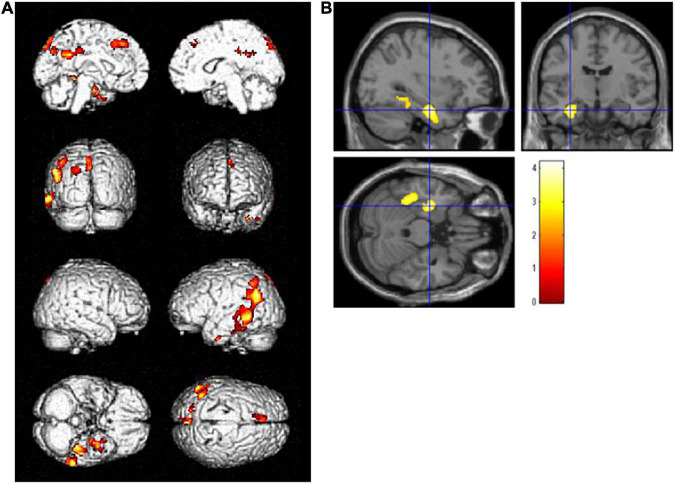
Regions where regional glucose metabolism was inversely associated with years of school education upon controlling for the Alzheimer’s Disease Assessment Scale-Cognitive Component-Japanese version score in a combined group (*N* = 57) of high- and low-education group. Statistically significant areas (*p* < 0.01, extent threshold >400) are displayed on **(A)** the brain surface projection and **(B)** multiplanar cross-sectional views. The red-yellow scale indicates the level of statistical significance. The blue crossed lines indicate the maximum peak voxel in the left hippocampus [(–32, –11, –24), *T* = 4.20].

**FIGURE 3:**
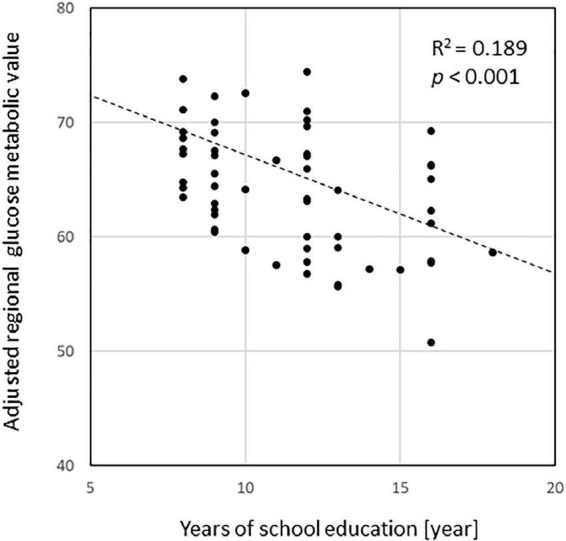
A scatterplot representing a significant inverse relationship (*R*^2^ = 0.189, *p* < 0.001) between the years of education (*x*-axis) and regional cerebral glucose metabolism in the left hippocampal cluster detected in the regression analysis (*y*-axis). Glucose metabolism values were normalized with the mean value in individual images as 50.

Voxel-based regression analyses controlling the ADAS-Jcog score revealed that years of school education were significantly and inversely associated with the regional glucose metabolism in the left hippocampus [(−32, −11, −24), *T* = 4.20] and the temporoparietal area, including the fusiform, inferior temporal, angular, and superior parietal gyrus. The left lingual and superior frontal gyri were also detected as significant areas [*p* < 0.01, extent threshold >400 (uncorrected)] (Figure 2). Figure 3 shows a scatter plot representing a significant inverse relationship (*R*^2^ = 0.189, *p* < 0.001) between years of school education (*x*-axis) and regional cerebral glucose metabolism in the left hippocampal cluster (*y*-axis).

**FIGURE 4:**
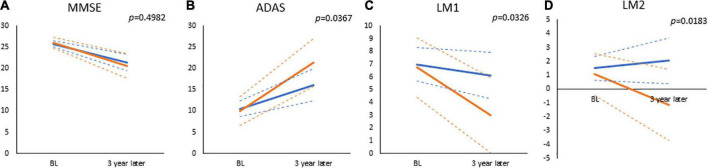
The results of general mixed linear models, which estimated values at baseline and visit of 3-year follow-up for **(A)** Mini-Mental State Examination (MMSE), **(B)** Alzheimer’s Disease Assessment Scale-Cognitive Component-Japanese version (ADAS), **(C)** Wechsler Memory Scale-Revised, logical memory I (LM1), and **(D)** Wechsler Memory Scale-Revised, logical memory II (LM2). Displayed *p*-values are significant for the interaction of time and education (high or low cognitive reserve). The solid and dashed lines connect the mean and 95% confidence interval at baseline and 3 years, respectively. Blue lines: low-education group (years of school education ≤12); Red lines: high-education group (years of school education ≥13).

Table 2 and Figure 4 show the results of the analyses of the GLMMs. The term for education was not significant for any of the subtests (MMSE, β = −0.3080, *p* = 0.6172; ADAS-Jcog, β = 0.5188, *p* = 0.7489; WMS-R LM I, β = 0.2624, *p* = 0.8182; WMS-R LM II, β = 0.4242, *p* = 0.5665), which represents no significance in any of the estimated cognitive scores between high- and low-education groups at the baseline. The term for time was significant for all the subtests (MMSE, β = −1.8162, *p* < 0.0001; ADAS-Jcog, β = 3.7829, *p* < 0.0001; WMS-R LM I, β = −1.2465, *p* = 0.0012; WMS-R LM II, β = −0.7317, *p* = 0.0240), which shows that significant cognitive decline over 3 years was estimated for all the cognitive scores. Education × time, which was the interaction effect of education and time, was significant for all the subtests except MMSE (MMSE, β = 0.3627, *p* = 0.4982; ADAS-Jcog, β = −1.9065, *p* = 0.0367; WMS-R LM I, β = 0.9491, *p* = 0.0326; WMS-R LM II, β = 0.9133, *p* = 0.0183).

**TABLE 2:** Parameter values estimated by the mixed general linear model.

	Model terms	Parameter estimate	SE	*P*-Value
MMSE	Education	−0.3080	0.6142	0.6172
	Time	−1.8162	0.4423	<0.0001
	Education × time	0.3627	0.5333	0.4982
ADAS-Jcog	Education	0.5188	1.6151	0.7489
	Time	3.7829	0.7218	<0.0001
	Education × time	−1.9065	0.8970	0.0367
WMS-R LM I	Education	0.2624	1.1380	0.8182
	Time	−1.2465	0.3643	0.0012
	Education × time	0.9491	0.4397	0.0326
WMS-R LM II	Education	0.4242	0.7373	0.5665
	Time	−0.7317	0.3151	0.0240
	Education × time	0.9133	0.3796	0.0183

Higher scores indicate better performance in the MMSE, LM I, and LM II and lower performance in the ADAS-Jcog. Possible scores for MMSE are 0–30; ADAS-Jcog, 0–70; LM-I, 0–50; and LM-II, 50. Time = years since baseline, Education = 0 [high-education: reference (years of school education ≥13) or 1 (low-education, years of school education ≤12)]. In addition to the variables shown in the table, each model included terms to control the fixed effects of age at baseline, sex as a covariate, and the random effects of the intercept (baseline performance) and slope (change over time). The estimated parameters are the effect of education at baseline (education), effect of elapsed time (time), and effect of interaction of education and time (education × time). MMSE, Mini-Mental State Examination; ADAS-Jcog, Alzheimer’s Disease Assessment Scale-Cognitive Component-Japanese version; WMS-R LM-I, Wechsler Memory Scale-Revised, logical memory I; WMS-R LM-II, Wechsler Memory Scale-Revised, logical memory II; SE, standard error of mean.

## Discussion

This study examined the effect of CR on amnestic MCI due to AD with neurodegeneration, as determined by FDG-PET.

All 57 patients with amnestic MCI included in this study showed AD-like hypometabolic pattern on FDG-PET images. They were not tested for amyloid by PET, CSF, or blood. Therefore, they can be categorized as MCI due to AD-intermediate likelihood based on the 2011 MCI criteria by the National Institute on Aging and Alzheimer’s Association (Albert et al., 2011).

Fluorodeoxyglucose-positron emission tomography is a downstream marker of neurodegeneration in AD (Dubois et al., 2014) that corresponds to N+ in ATN system in AD continuum (Jack et al., 2016). Although the topographic pattern of hypometabolism in the parietotemporal association cortex, posterior cingulate gyrus, and precuneus is useful for diagnosing AD and may be an alternative to amyloid PET in some conditions (Rabinovici et al., 2011), such a pattern can be observed in neurological diseases other than AD (Kato et al., 2016). Tau PET and amyloid PET would provide all the information on ATN and would allow a more reliable and detailed evaluation of the CR of amnestic MCI in the AD continuum.

Years of school education was used as a surrogate index of CR for this study. In the statistical group comparison adjusting for the cognitive performance, significantly more severe hypometabolism was detected in the left hippocampus and surrounding regions, including the fusiform and parahippocampal gyrus, in the high-education group than in the low-education group (Figure 1). In addition, areas where regional glucose metabolism was negatively correlated with years of school education adjusting for the cognitive score were found in the left temporal to parietal lobes (Figure 2). In both analyses, the clusters of the left hippocampal regions had the highest *T*-values. These brain regions are within the area where tau accumulation and neurodegeneration are observed in AD. These results revealed that in amnestic MCI due to AD, if cognitive function is set at the same level, the longer years of education are associated with more advanced neurodegeneration of AD. The concept of CR relates to the capacity of the brain to cope with neuropathology so as to minimize clinical manifestations in aging and dementia (Stern, 2002). Accordingly, we can reason out that the CR effect manifests as a decline in the cerebral glucose metabolism in amnestic MCI due to AD with the same level of cognition.

The cerebral regions related to CR were distributed in a strong left-dominant manner, which was not seen in the z-score mapping of mean FDG-PET images showing the P1 pattern (Ito et al., 2015). This may be explained by the fact that the performance of language-based neuropsychological tests has been shown to correlate with the lateralization of gray matter loss to the left hemisphere in MCI and AD (Derflinger et al., 2011).

This study also examined the effect of educational attainment on the estimated rate of cognitive decline during a 3-year follow-up period using GLMMs. An interaction effect of years of school education on the cognitive decline rate was found for the ADAS-Jcog, LM-I, and LM-II (Table 2 and Figure 4). These results indicate that cognitive decline following the onset of AD is faster for those with a high-education than for those with low-education. No significant difference in the cognitive function at baseline was observed between the high- and low-education groups. This finding could be attributed to the inclusion criteria, which encompassed amnestic MCI and a combination of MMSE, WMS-R-LM-I/II, CDR, and GDS scores. The narrow range of cognitive scores may have resulted in the inclusion of amnestic MCI with relatively uniform cognitive function scores. If the inclusion criteria had been more relaxed, significant differences caused by CR between the high- and low-education groups might have been detected. The rates of cognitive decline of the patients might have been influenced by the difference of cognitive status.

Recently, biomarkers reflecting amyloid and tau, which are involved in the pathology of AD, have become available. It has become possible to narrow the focus of CR studies to AD continuum or preclinical AD by incorporating these biomarkers. The results of these studies have indicated that the rate of cognitive decline in the high-education group is slower in the preclinical AD stage and faster after the onset of AD (van Loenhoud et al., 2019; Lee et al., 2021). The results of this study were consistent with the above findings.

Epidemiological studies examining CR suggest that CR is linked to socioeconomic status and literacy (Stern, 2009). Years of schooling is a strong proxy for CR, but it is not a decisive factor. Years of schooling are correlated with family environment and general socioeconomic attainment. Therefore, the characteristics of years of schooling as a proxy indicator of CR may vary based on conditions, such as race and social environment. In the United States, the effect of years of education reportedly varies by race (Avila et al., 2020). This could be attributed to the social and economic environment in which the people of a particular race live, rather than a factor of race itself. The participants of this study were Japanese individuals born during or shortly after World War II. College and university enrollment rates among this population were generally around 10%, suggesting that most individuals with 13 or more years of education (i.e., more than secondary schooling) belonged to a relatively high social and economic stratum. The division between up to and beyond secondary education is found in several CR studies (Nishita et al., 2019; Wilson et al., 2019; Avila et al., 2020). The appropriate separating point for educational length may vary based on the distribution of the years of education and socioeconomic background of the studied population. It may be useful to utilize sensitivity analysis (Qian and Mahdi, 2020) to optimize the split points. Additional analyses dividing the subjects into the compulsory education level (years of school education ≤9, *N* = 21) and higher education level (years of school education ≥13, *N* = 18) provided nearly identical results that are shown in the Supplementary Materials 3A–E. The general direction that the results of the present analyses represent is maintained.

We should be cautious about the extent to which results of this study can be generalized. These results may not be equally applicable to subjects with different characteristics using different analysis methods (Lovden et al., 2020). The findings of this study are for patients with amnestic MCI due to AD-intermediate likelihood with a narrow range of cognitive scores and an average age of approximately 71 years. Younger age may have increased the proportion of hippocampal-sparing AD type, while older age may have raised the proportion of limbic predominant AD subtype (Ferreira et al., 2020). These subtypes differ in terms of the affected brain regions and the degree of atrophy, as well as in the rate of progression of cognitive decline. With increasing age, more pathological changes other than AD become evident (Jellinger, 1998; Spina et al., 2021). Even if diagnosis of AD is narrowed using imaging or fluid biomarkers, the results may differ from this study depending on the characteristics of the recruited subjects. The same may be true for targeting non-AD dementias. The measured cognitive scores should also be considered. In a cohort study with a larger sample size, the higher education group demonstrated accelerated declines in composite scores of episodic memory and perceptual speed, but not in the composite score of global cognition (Wilson et al., 2019).

The other potential factors such as sleeping disturbance (Cordone et al., 2019), ApoE type (Vemuri et al., 2017), physical activity (Pedrero-Chamizo et al., 2021), and cerebrovascular disease (Saito and Ihara, 2016) are ideally also to be analyzed, because they may be linked to disease progression through amyloid and tau deposition or other pathways. Patients with cerebrovascular disease were excluded in this study. Unfortunately, ApoE type, sleeping disorder, and physical activities were not evaluated. It is expected that such modifying factors will be examined in the future.

This study has certain limitations. First, the sample size was relatively small. As the significance level of the present results is not sufficiently high, a study with a larger sample size is necessary to obtain more robust results. Second, there was a lack of biomarker information directly indicating the presence or absence of amyloid and tau. Third, information on *ApoE* was not available; *ApoE* is a risk factor for AD, which could result in a bias in the present findings. Finally, the assessment measures could also be a limitation of the study. We used years of education as a proxy measure of CR. CR is defined not only by years of education but also by socioeconomic activities, language proficiency, leisure activity level, and physical activity level. The use of a composite proxy index that includes these factors could result in a more accurate assessment (Ko et al., 2022). Considering cognitive function, this study examined limited cognitive function scores, such as the ADAS-Jcog; however, previous studies have demonstrated that the CR effect varies based on the cognitive function (Kang et al., 2018). It might be desirable to evaluate various brain functions, including executive functions, individually and as a combination.

In conclusion, this study demonstrated that cerebral glucose metabolism is lower and cognitive function declines faster in patients with high CR in amnestic MCI due to AD defined by FDG-PET.

## Data availability statement

The dataset can be requested from the principal investigator upon reasonable request. Requests to access the datasets should be directed to TK, tkato@ncgg.go.jp.

## Ethics statement

The studies involving human participants were reviewed and approved by the Ethics Committee of the National Center for Geriatrics and Gerontology. The patients/participants provided their written informed consent to participate in this study.

## Members of SEAD-J Study Group

Hidenao Fukuyama (Kyoto University, Kyoto, Japan), Michio Senda (Institute of Biomedical Research and Innovation, Kobe, Japan), Kenji Ishii (Tokyo Metropolitan Institute of Gerontology, Tokyo, Japan), Kazunari Ishii (Kindai University, Osaka, Japan), Kiyoshi Maeda (Kobe Gakuin University, Kobe, Japan), Yasuji Yamamoto (Kobe University Graduate School of Medicine, Kobe, Japan), Yasuomi Ouchi (Hamamatsu University School of Medicine, Hamamatsu, Japan), Ayumu Okamura (Kizawa Memorial Hospital, Gifu, Japan), Yutaka Arahata (National Center for Geriatrics and Gerontology, Aichi, Japan), Yukihiko Washimi (National Center for Geriatrics and Gerontology, Aichi, Japan), Kenichi Meguro (Tohoku University Graduate School of Medicine, Sendai, Japan), and Mitsuru Ikeda (Nagoya University School of Health Sciences, Nagoya, Japan).

## Author contributions

KI was the principal investigator who contributed to the design and administration of the SEAD-J study. YI made basic data preparation and analyses. TK performed voxel-based statistical analyses and wrote the first draft of the manuscript. YN and RO analyzed the general mixed linear models. YK and AN contributed to the structure and the discussion of the manuscript. All authors contributed to the article and approved the submitted version.

## Footnotes

1
http://marsbar.sourceforge.net/
