# Supplementary material for: Effect of cognitive reserve on amnestic mild cognitive impairment due to Alzheimer’s disease defined by fluorodeoxyglucose-positron emission tomography
Source: Front Aging Neurosci. 2022 Aug 10;14:932906. doi: 10.3389/fnagi.2022.932906 (PMC9399434; doi:10.3389/fnagi.2022.932906)
Supplement: Supplementary file 1 [file Data_Sheet_1.PDF]

**Supplementary materials 3(a)**

**Supplementary Table 1. Demographics of the patients at the baseline and conversion rate in 3-year follow-up. Lower education group (years of school education ≤9; N=21) and higher education group (years of school education ≥ 13; N=18)**

| | Total | Lower-education | High-education | *p* |
|---|---|---|---|---|
| N | 39 | 21 | 18 | |
| Age | 72.0 (6.8) | 74.2 (4.5) | 69.3 (8.0) | 0.022 |
| Sex (male/female) | 19/20 | 5/16 | 14/4 | 0.001 |
| Years of school education | 11.6 (3.5) | 8.6 (0.5) (≤12) | 15.1 (1.5 )(≥13) | <0.001 |
| CDR | 0.5 | 0.5 | 0.5 | |
| MMSE | 26.0 (1.6) | 25.9 (1.7) | 26.0 (1.6) | 0.858 |
| ADAS-Jcog | 10.8 (5.2) | 12.2 (6.0) | 9.2 (3.5) | 0.069 |
| WMS-R LM-I | 7.1 (3.0) | 7.0 (3.1) | 7.3 (3.0) | 0.779 |
| WMS-R LM-II | 2.4 (2.3) | 2.3 (2.3) | 2.4 (2.5) | 0.942 |
| GDS | 4.5 (2.2) | 4.2 (2.4) | 4.8 (2.0) | 0.457 |
| Converter/non-converter | 25/14 | 12/9 | 13/5 | 0.328 |

Values are presented as mean (standard deviation) or number of participants. Differences in sex and conversion/non-conversion between the high and low cognitive reserves were tested using the chi-square test. Other continuous parameters were tested using the student's t-test. CDR, Clinical Dementia Rating Scale; MMSE, Mini-Mental State Examination; ADAS-Jcog, Alzheimer's Disease Assessment Scale-Cognitive Component-Japanese version; WMS-R LM-I, Wechsler Memory Scale-Revised, logical memory I; WMS-R LM-II, Wechsler Memory Scale-Revised, logical memory II; GDS, Geriatric Depression Scale.
