# Supplementary material for: Effect of cognitive reserve on amnestic mild cognitive impairment due to Alzheimer’s disease defined by fluorodeoxyglucose-positron emission tomography
Source: Front Aging Neurosci. 2022 Aug 10;14:932906. doi: 10.3389/fnagi.2022.932906 (PMC9399434; doi:10.3389/fnagi.2022.932906)
Supplement: Supplementary file 2 [file Data_Sheet_2.PDF]

Supplementary materials 3(b)

Supplementary Table 2.| Parameter values estimated by the mixed general linear model in the data set of lower education group (years of school education ≤9; N=21) and higher education group (years of school education ≥ 13; N=18)

	Model terms	Parameter estimate	SE	*p*-value
MMSE	Education	0.01942	0.7727	0.9800
	Time	-1.8172	0.4430	0.0002
	Education × time	0.1866	0.5939	0.7544
ADAS-Jcog	Education	2.1047	2.1203	0.3251
	Time	3.8698	0.8036	<0.0001
	Education × time	-2.4346	1.1396	0.0370
WMS-R LM I	Education	-0.9007	1.4051	0.5238
	Time	-1.2441	0.3695	0.0018
	Education × time	1.2088	0.4905	0.0164
WMS-R LM II	Education	0.3992	0.8837	0.6530
	Time	-0.7260	0.2871	0.0158
	Education × time	0.9295	0.3819	0.0177

Note: Higher scores indicate better performance in the MMSE, LM I, and LM II and lower performance in the ADAS-Jcog. Possible scores for: MMSE, are 0–30; ADAS-Jcog, 0–70; LM-I, 0-50; and LM-II, 50. Time = years since baseline, Education = 0 (high-education: reference [years of school education ≥ 13] or 1 [low-education, years of school education ≤9]). In addition to the variables shown in the table, each model included terms to control the fixed effects of age at baseline, sex as a covariate, and the random effects of the intercept (baseline performance) and slope (change over time). The estimated parameters are the effect of education at baseline (education), effect of elapsed time (time), and effect of interaction of education and time (education × time). MMSE, Mini-Mental State Examination; ADAS-Jcog, Alzheimer's Disease Assessment Scale-Cognitive

Component-Japanese version; WMS-R LM-I, Wechsler Memory Scale-Revised, logical memory I; WMS-R LM-II, Wechsler Memory Scale-Revised, logical memory II; SE, standard error of mean.
