# Supplementary figures and images for: Effect of cognitive reserve on amnestic mild cognitive impairment due to Alzheimer’s disease defined by fluorodeoxyglucose-positron emission tomography
Source: Front Aging Neurosci. 2022 Aug 10;14:932906. doi: 10.3389/fnagi.2022.932906 (PMC9399434; doi:10.3389/fnagi.2022.932906)

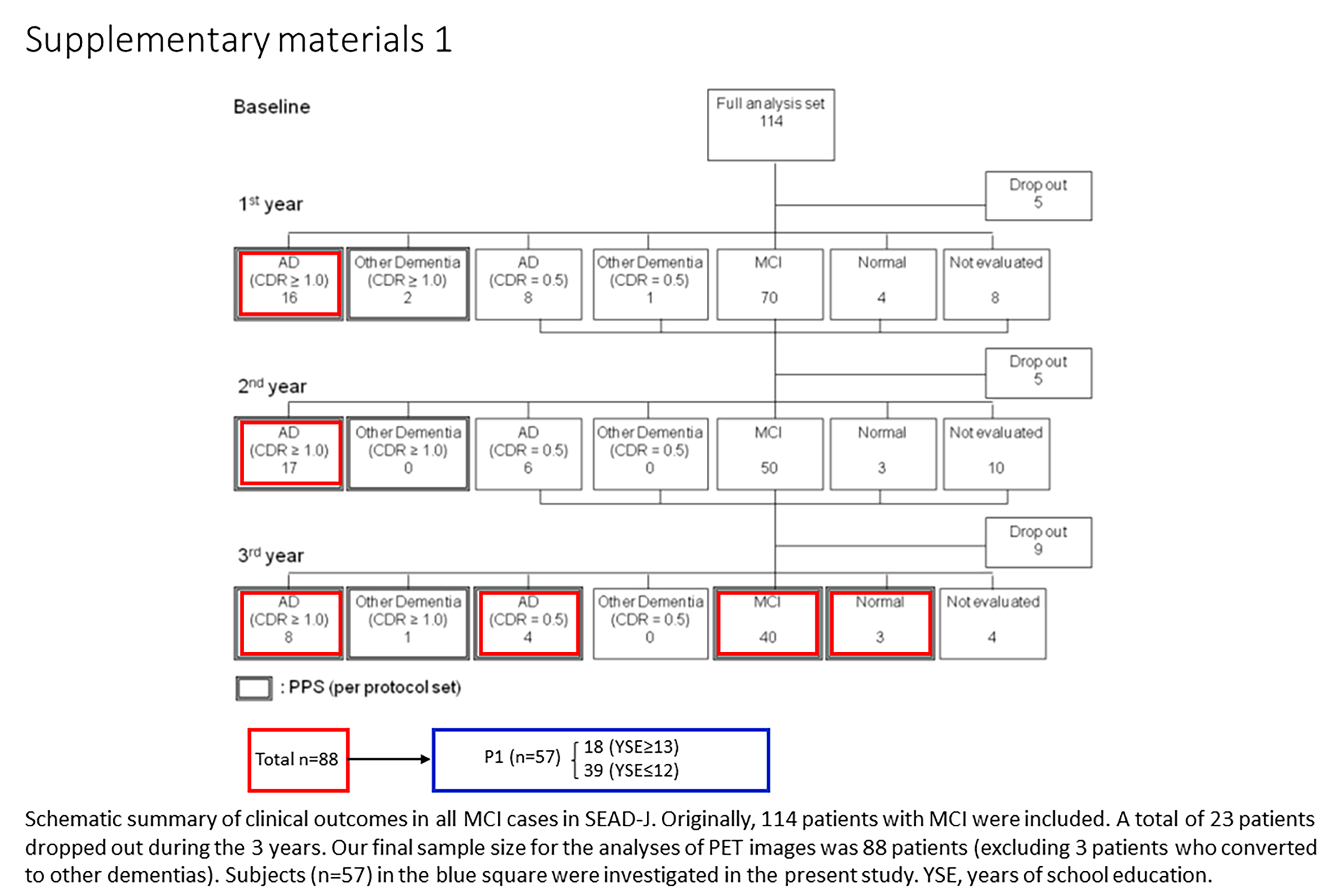

Supplement: Supplementary file 3 [file Image_1.TIF]

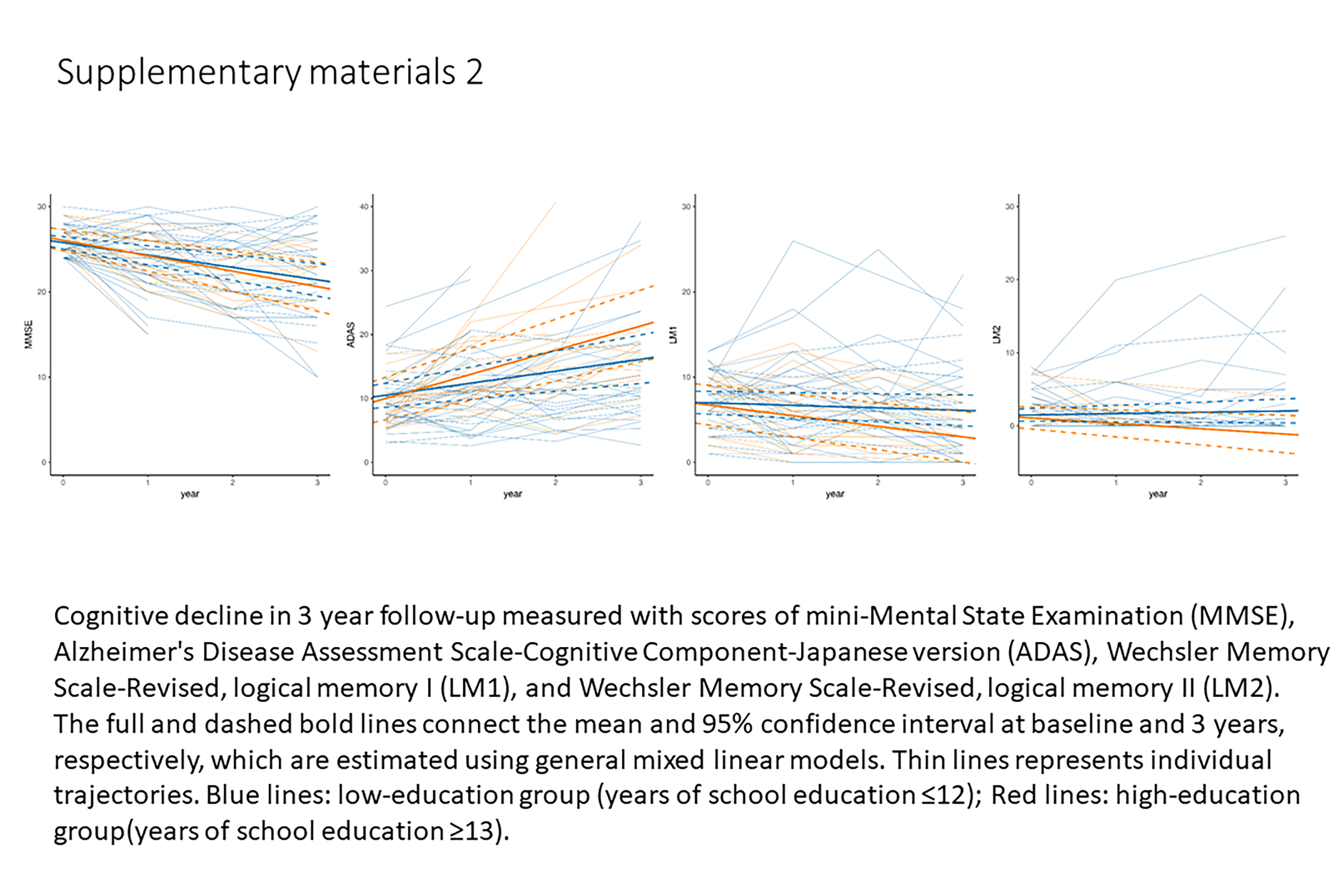

Supplement: Supplementary file 4 [file Image_2.TIF]

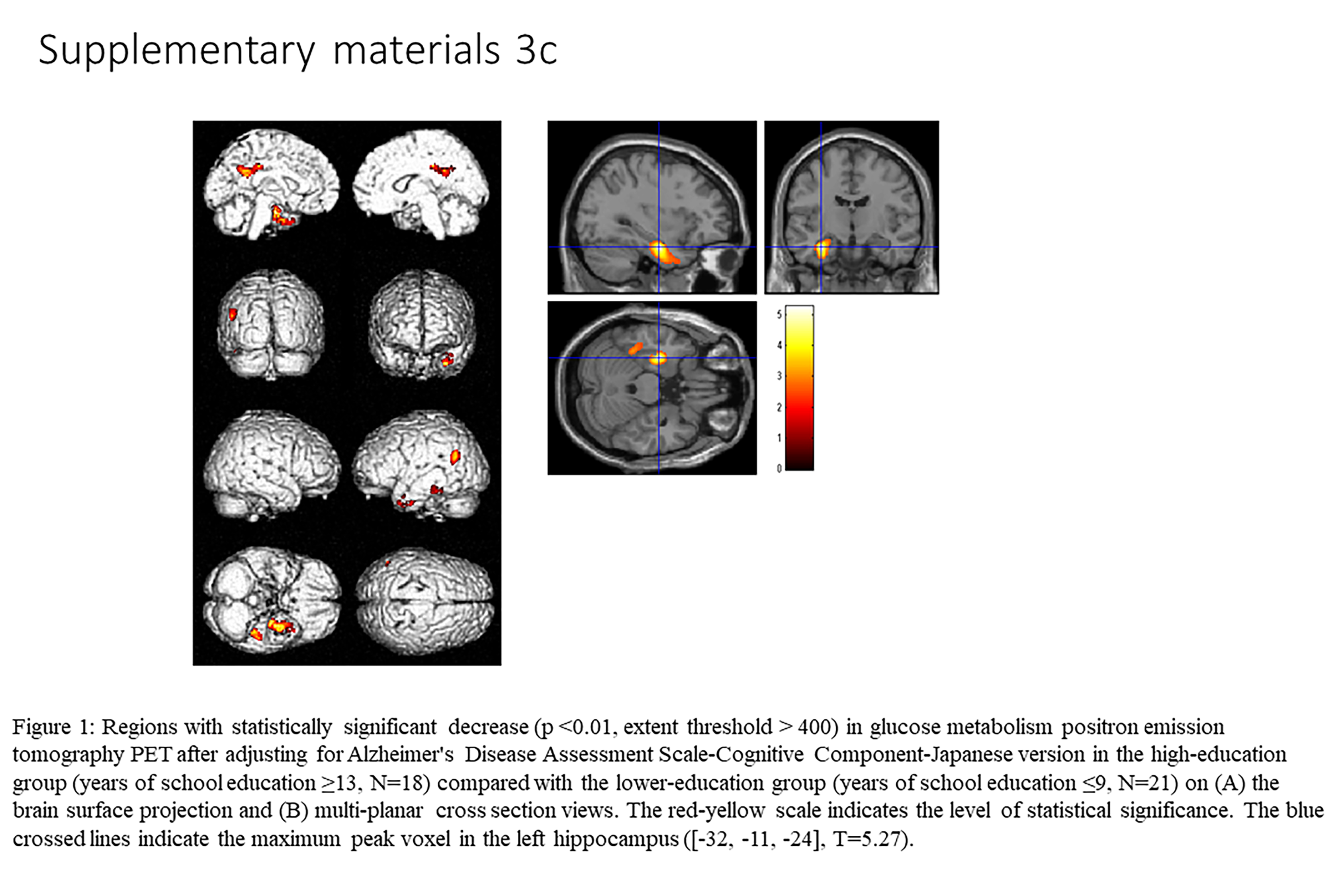

Supplement: Supplementary file 5 [file Image_3.TIF]

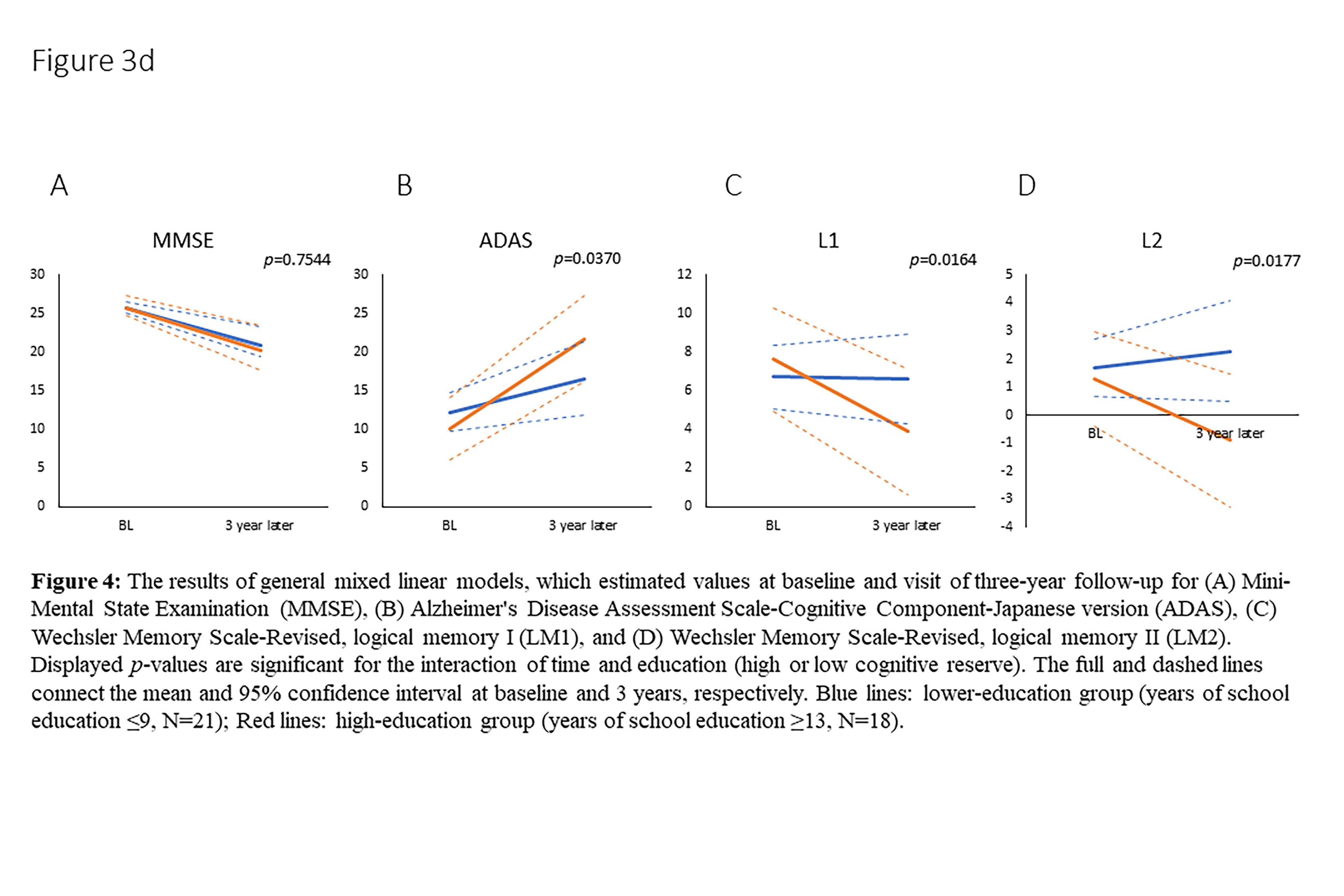

Supplement: Supplementary file 6 [file Image_4.TIF]

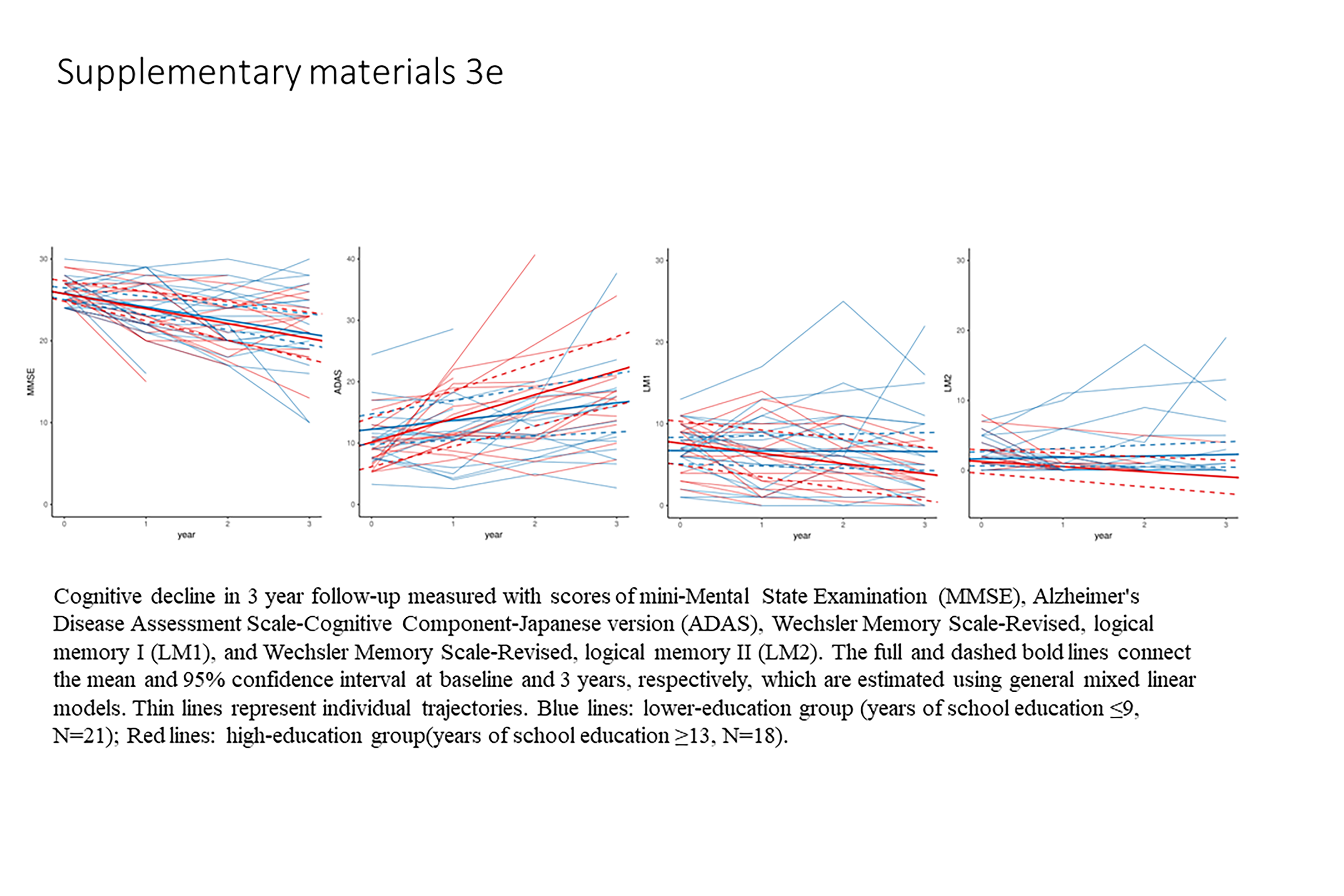

Supplement: Supplementary file 7 [file Image_5.TIF]
